# Postpartum Anorectal and Pelvic Floor Disorders: Evaluation, Treatment, and Prevention

**DOI:** 10.1007/s11894-025-01000-7

**Published:** 2025-07-03

**Authors:** Lalitha Sitaraman, Christina Lewicky-Gaupp, Satish SC Rao

**Affiliations:** 1 Section of Gastroenterology, Hepatology, and Nutrition, University of Chicago, 5841 S. Maryland Ave, MC 4080, Chicago, IL 60637 USA; 2 Department of Obstetrics and Gynecology, Section of Urogynecology and Reconstructive Pelvic Surgery, University of Chicago, Chicago, IL USA; 3 Division of Gastroenterology and Hepatology, Augusta University, Augusta, GA USA

**Keywords:** Postpartum, Fecal incontinence, Obstetric anal sphincter injury, Pelvic floor disorder, Anorectal disorder

## Abstract

**Purpose of Review:**

Postpartum anorectal and pelvic floor disorders (PFD) are common, though under-recognized. There is limited knowledge regarding their diagnosis, treatment, and prevention. Here we provide a critical review of this topic and highlight knowledge gaps and treatment options for these problems.

**Recent Findings:**

Recent advances include dynamic 3D and 4D ultrasound of the pelvic floor to reveal pathology, anal sphincter defects, and pelvic organ prolapse. Treatments for fecal incontinence include anal inserts, vaginal inserts, translumbosacral neuromodulation therapy (not yet studied postpartum), and increasing data for safety of sacral nerve stimulators in pregnancy. Exercise, pelvic floor muscle training, and use of special devices show mixed results.

**Summary:**

Postpartum anorectal and pelvic floor disorders include fecal and/or flatus incontinence, constipation, hemorrhoids, pelvic organ prolapse, and urinary incontinence. Many patients present years later, and most suffer in silence. Early recognition, appropriate treatment, and preventative measures could mitigate these problems.

**Supplementary Information:**

The online version contains supplementary material available at 10.1007/s11894-025-01000-7.

## Introduction

It is well-known that pregnancy and childbirth affect the pelvic floor. Common postpartum problems include urinary incontinence, pelvic organ prolapse, and sexual dysfunction, in addition to anorectal complaints such as fecal incontinence, constipation, and hemorrhoids [1–3]. Their prevalence rates vary widely, but are under reported due to embarrassment, lack of screening, assessment methods, and others [4–6]. Recommendations for treatment and preventative strategies are based on low quality evidence, with an overall paucity of well-designed studies. The goals of this review are to present the available data on postpartum anorectal and pelvic floor disorders (PFD), highlight the gaps in knowledge, and propose strategies for future research.

Anatomically, the female perineum and pelvic floor can be divided into the urogenital triangle, perineal body, anal triangle, and levator ani muscle complex. Simplified, the urogenital triangle is comprised of the dorsal region (vagina) and ventral region (urethra and clitoris). The anal triangle contains the anal canal, the internal and external anal sphincters, and the ischioanal fossa. The perineal body is the central point between the urogenital and anal triangles of the perineum [7]. The levator ani complex consists of three muscles: iliococcygeous, pubococcygeous, and puborectalis. The levator ani forms the floor of the pelvis and supports the viscera of the pelvic cavity, maintains continence, aids in defecation, and plays a role in sexual function [7, 8]. 

During pregnancy, the hormone progesterone causes smooth muscle to relax, to prevent premature uterine contractions. Progesterone also affects the gut smooth muscle, causing delayed gastric emptying, constipation, and diminished lower esophageal sphincter and anal sphincter tone [8]. Musculoskeletal changes also occur during pregnancy including ligamentous laxity allowing the pelvis to stretch and accommodate the gravid uterus [7, 8]. However, this laxity may ultimately lead to pathology [8]. Additionally, the hormone relaxin, which relaxes the symphysis pubis and cervix during pregnancy, decreases ileal smooth muscle contractions, further contributing to constipation in pregnancy and slowing of small bowel transit [9]. 

Labor and delivery of the fetus can have complications with long-term consequences. A common complication of vaginal birth is a perineal tear, graded on a scale of first to fourth degree (Table 1) [10]. Second-degree tears are the most common type of tear and are presumed to heal on their own once sutured [11]. Third- and fourth-degree tears are grouped together as obstetric anal sphincter injuries (OASIS) [10]. Episiotomy, a surgical incision to increase the diameter of the vulval outlet, used to be more frequently performed to prevent perineal trauma [7]. However, episiotomy is now avoided, as all evidence has shown that episiotomy is one of the most significant risk factors for OASIS [12]. Other risk factors for OASIS are well-established, including prolonged second stage of delivery, use of epidural, forceps or vacuum assisted delivery, and birth weight more than 4 kg [4]. Protective factors for OASIS include perineal massage starting at 34 weeks gestation, restricted mediolateral episiotomy, and perineal massage and warm compresses on the perineum during the second stage of labor [5]. However, patients may experience PFD including urinary incontinence (UI), fecal incontinence (FI), and pelvic organ prolapse (POP), even without OASIS or with Cesarian delivery [5, 11]. 

**Table 1:** Classification of perineal tears

Classification	Injury
First Degree	Injury to perineal skin and/or vaginal mucosa
Second Degree	Injury to perineum involving perineal muscles but not involving the anal sphincter.
Third Degree	Injury to perineum involving the anal sphincter complex
3a	Less than 50% of external anal sphincter (EAS) thickness torn
3b	More than 50% of EAS thickness torn
3c	Both EAS and internal anal sphincter (IAS) torn.
Fourth Degree	Injury to perineum involving the anal sphincter complex (EAS and IAS) and anorectal mucosa.

Several questionnaires have been developed to standardize symptom assessment and quantify the impact of PFD symptoms on quality of life. In 2001, two questionnaires were developed for all forms of female PFD: the Pelvic Floor Distress Inventory (PFDI) and the Pelvic Floor Impact Questionnaire (PFIQ) [13]. In 2004, short forms of these questionnaires were developed, with the same validity and reliability: The Pelvic Floor Distress Inventory-20 and Pelvic Floor Impact Questionnaire-7. (Supplement 1) [14] The PFDI-20 quantifies bother of urinary, colorectal/anal, and POP symptoms, and PFIQ-7 assesses impact of symptoms on quality of life; these are the most widely utilized [15]. However, these questionnaires have not been validated in the post-partum population.

### Fecal and Flatus Incontinence

Frequently termed “anal incontinence” in the literature, the more correct terminology is specifying fecal incontinence and/or flatus incontinence. For brevity, FI will be used to describe incontinence of feces and flatus. FI prevalence varies in the literature, especially when stool and gas leakage are separated and the time after delivery is considered. Rates reported for FI are as low as 7% and as high as 25% in the peripartum period [2, 4, 8, 16]. The frequency of flatus incontinence was nearly 40% in women with OASIS [4]. Rates of FI are highest immediately postpartum and decrease over time, though can be as high as 20% at 1 year [2, 16]. 

However, these rates may even be underreported, due to the embarrassment associated with FI; women who seek medical care for FI has been reported as being less than 15% [8]. FI is associated with poor quality of life, including loss of confidence and self-respect, compounded by the social stigma attached to FI [17]. Providers also do not routinely screen for FI, including women in routine gynecologic care [17]. 

Known etiologies and risk factors for postpartum FI include vaginal delivery, operative vaginal delivery, any perineal laceration (especially OASIS), increasing parity, FI during pregnancy, and obstructed defecation [2, 8, 18]. 

#### Diagnostic Tools

There are multiple methods to evaluate the etiology of FI. The first tool to assess FI is a digital rectal exam. Evaluation of the perineal body and evaluation for a circumferential squeeze is essential to determine if there is a sphincter defect [2, 19]. Anal ultrasound is helpful for surgical planning when a sphincter defect is suspected [2, 20]. 

**Fig. 1:**
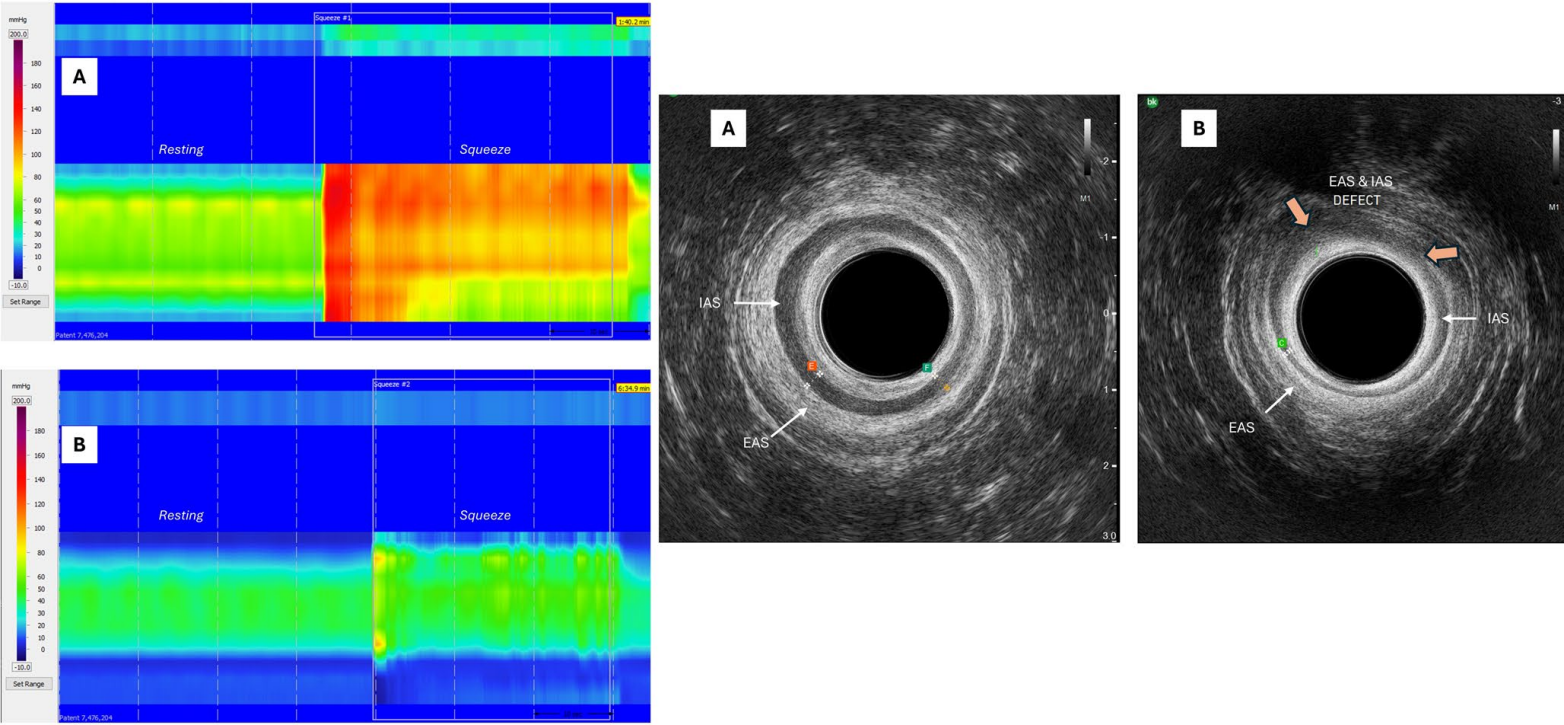
Left: High resolution anorectal manometry resting pressures and squeeze profile in a healthy patient (**A**) and a patient with fecal incontinence (**B**). Right: Anal ultrasound in a healthy patient (**A**) and patient with postpartum anal sphincter injury (**B**)

Anorectal manometry can objectively determine low resting tone (primarily internal anal sphincter) and low squeeze pressure (primarily external anal sphincter) [19, 21]. Other findings on anorectal manometry that can signal FI include an abnormal cough reflex, rectal hyposensitivity or hypersensitivity, and impaired rectal compliance [21]. Abnormal cough reflex or changes in sensation are suggestive of neurologic abnormalities [21]. Examples of anorectal manometry and anal ultrasound findings are shown in Fig. 1.

If there is posterior vaginal prolapse (formerly termed a “rectocele”), this may also influence surgical planning in the setting of an external anal sphincteroplasty [20]. Diagnosis of posterior vaginal prolapse is best evaluated clinically [22]. Dynamic imaging of defecation, such as with MR or barium defecography, in addition to dynamic ultrasound can also be used to support the diagnosis [19, 23]. Dynamic pelvic floor ultrasound has 3 commonly used modalities: endoanal/endorectal (aPFUS), transperineal/introital (pPFUS), and endovaginal (vPFUS). Concordance of findings with defecography and dynamic ultrasound has been validated, with good accuracy [23]. Ultrasound has also been studied in postpartum PFD to diagnose and follow post-treatment. In the study by Zhang et al., four-dimensional ultrasound was performed to identify dynamic changes in structure and function of areas such as bladder neck descent, levator ani thickness under rest state, levator ani thickness under Valsalva, among others. The study found increased measurements of all areas evaluated in those with PFD compared to controls, and in patients who did not recover after treatment, these measurements remained elevated [24]. This type of ultrasound shows promise for diagnostic and prognostic purposes.

In the general population, any patient with FI should have considerations of other causes, including fecal impaction, chronic diarrhea, and malignancy. This does not exclude the postpartum patient population. Colonoscopy is usually not necessary for the evaluation of FI, though recommendations of its routine use among experts differs [19, 20, 25, 26]. 

#### Treatment

##### Medical Treatment

Dietary modifications include increasing fiber intake, limiting caffeine, alcohol, and fatty foods [19]. Antidiarrheals may help 15% of patients. In some cases, a diet low in FODMAPs (fermentable oligo-, di-, and mono-saccharides and polyols) may be helpful, if patients have diarrhea or urgency associated with high FODMAP foods [20, 25]. Fiber supplements such as psyllium and gum arabic improve FI [20, 25]. Lifestyle modifications recommended include timed toileting and urgency training [20, 25, 26]. In the general population, weight loss and quitting smoking may also have a positive effect on FI [19]. 

##### Behavioral Training

Biofeedback therapy with a focus on strengthening the anal sphincters and pelvic floor is a common recommendation [2]. During biofeedback, patients receive visual signals of bodily activity, such as anorectal pressure during squeeze. Additionally, pelvic floor muscle therapy (PFMT) may help. Although the superiority of biofeedback compared to medical therapy or FI education remains unclear, a randomized controlled trial showed biofeedback was more effective than PFMT alone [27–29].

##### Vaginal Electrical Stimulation (e-stim)

This modality has been utilized alone and in conjunction with PFMT and biofeedback. Results are mixed. In one postpartum study, vaginal e-stim paradoxically increased FI symptoms [30]. In a study of biofeedback coupled with e-stim, while intra-anal electromyographic biofeedback therapy was associated with improved continence and quality of life in women with FI after delivery, the addition of e-stim did not enhance symptomatic outcomes [31]. 

##### Other Nonsurgical Methods 

Mechanical insert/barrier devices and perianal injection of bulking agents are useful but not yet studied in the immediate postpartum period. The anal insert (Renew insert, Renew Medical, Foster City, CA) and a vaginal bowel-control system (Eclipse System, Pelvalon Inc, Sunnyvale CA) showed promise in open-label trials [19]. While the Eclipse device can be utilized postpartum, it is imperative to ensure that the vagina has healed prior to insertion. The most common injectable medication is dextranomer microspheres stabilized with hyaluronic acid (NASHA/Dx, Solesta, Palette Life Sciences, Santa Barbara, CA), although other injectable materials have been used. Studies have varying results, but overall are positive with 50% reduction of FI events [19, 20, 25]. 

##### Neuromodulation

Sacral nerve stimulation (SNS) or sacral neuromodulation (SNM) is successful in approximately 50% at 5–10 years in large observational cohort studies, although these numbers depend on the method of reporting and do not stratify results based on age or postpartum status [19]. SNS remains costly for some, though insurance coverage is improving. Maintenance of the device, including programming and battery replacement, was more cumbersome in the past [19, 25]. However, with improvement in battery life (upwards of 20 years per battery), as well as the innovation of batteries that only need recharging twice a year through the skin, SNS is a favored option for many postpartum patients who are refractory to other therapies. SNS can also be used in pregnancy. In a 2023 systematic review, SNM activation was safe and effective in pregnancy and it may be recommended on an individual basis [32, 33]. Another option for neuromodulation is percutaneous posterior tibial nerve stimulation, though it is not as effective as SNS in most studies [25]. A novel method is translumbosacral neuromodulation therapy (TNT) that targets neuropathy using low frequency repetitive magnetic stimulation [34]. Using translumbosacral anorectal magnetic stimulation test, up to 88% of FI patients were found to have prolonged latency of motor evoked potentials suggesting neuropathy and a rationale for TNT therapy [37].

##### Sphincteroplasty 

For patients with sphincter defects despite initial OASIS repair in the delivery room, sphincteroplasty is recommended. Timing of repair can be earlier than historically recommended; most data regarding timing of repair are published in case series. The most recent series examined 18 women with OASIS breakdown; median time from diagnosis of the OASIS breakdown and secondary operative revision was 19.5 days (interquartile range, 12-26.8 days). At three months postop, no woman had a recurrent breakdown or fistula [35]. However, long-term outcome studies of postpartum women who have undergone OASIS revision are lacking. In the literature, success rates decrease over time after sphincteroplasty, but the majority remain satisfied with their results. Outcomes of sphincteroplasty are worse when performed more than 10 years after initial trauma [2]. Other surgical options may be indicated if there is POP [19]. 

#### Prevention

There are no methods that have been proven to prevent postpartum FI. In a recent Cochrane analysis of PFMT and FI, eight trials reported FI outcomes. Overall, the data are low quality. In women with or without FI, there was no difference in the prevalence of FI in late pregnancy with antenatal PFMT (RR 0.64, 95% CI 0.36 to 1.14). Similarly, for postnatal PFMT in a mixed population, there was no evidence that PFMT reduces the risk of FI in the late postnatal period (RR 0.73, 95% CI 0.13 to 4.21) [16]. 

Cesarian delivery has not been proven to prevent FI compared to vaginal delivery. In a Cochrane review of 21 eligible studies, encompassing 31,698 women having had 6,028 Cesarian deliveries and 25,170 vaginal deliveries, and only one study showed a difference. However, their incontinence rates were extremely high, 39% in Cesarian and 48% in vaginal delivery, which questions the timing and nature of continence assessment, relative to other reports [36]. A randomized controlled trial was performed to determine whether planned Cesarean section for a second delivery protects against FI in women who previously had OASIS but were asymptomatic. Cesarean section for the second delivery did not protect against FI [37]. However, there is evidence that the risk of FI is lower in patients who do not have a vaginal birth, especially an operative one. In a seminal study, Nygaard et al. found that FI occurs in a large number of women 30 years after delivery, regardless of the type of delivery [38]. 

### Constipation

Postpartum constipation is extremely common, affecting 45% of women and is multifactorial [39]. In the acute postpartum period, lack of oral intake causing dehydration, use of magnesium sulfate to prevent preterm labor or preeclampsia, and use of opioids during labor may be the leading causes. However, pain of the perineum, with or without repaired episiotomy or perineal tear, and pain from Cesarean section could lead to hesitation to defecate as well [39]. Elevated progesterone levels from pregnancy can be associated with constipation in the weeks following pregnancy [8, 39]. 

The development of chronic constipation or dyssynergic defecation postpartum has not been described. Many women with irritable bowel syndrome (IBS) are of child-bearing age, and anecdotally, IBS may remit during pregnancy, only to recur postpartum [40]. Presumably, perineal pain could lead to dyssynergia. However, this has not been studied.

#### Treatment and Prevention

There is poor evidence for treatment and prevention of postpartum constipation. In a Cochrane review analyzing safety and efficacy of laxatives in postpartum patients from 2020, five trials involving a total of 1208 women were included; however, the trials were poorly designed, and 4 out of 5 trials were published more than 40 years ago [39]. Guidelines for constipation postpartum are nearly non-existent; the World Health Organization is the only major society that provides a recommendation, which includes nonspecific dietary advice [41]. In real world practice, a retrospective study showed considerable variation in prescriptions after OASIS, with the least evidence-based medications being most commonly used (docusate and psyllium) [42]. There are no trials that have assessed high fiber diet or exercise to prevent constipation postpartum [39]. 

### Hemorrhoids

Hemorrhoids commonly develop during pregnancy, usually in the third trimester, because of the enlarging uterus causing vascular engorgement, venous stasis, and increased intra-abdominal pressure, with up to 85% of pregnant persons reporting symptoms of hemorrhoids and anal fissures [3, 9]. In a recent study by Boughton, et al., nearly half of their cohort had symptoms of hemorrhoids or anal fissure, but the majority (61%) self-diagnosed and managed on their own without in the input of medical professionals [3]. They demonstrated resolution or improvement in 45% within a few days of delivery. Others have reported ongoing symptoms for 1.5 years postpartum [43]. 

#### Diagnosis

Internal hemorrhoids are graded by prolapse: no prolapse (first degree); prolapse on straining and spontaneous reduction (second degree); prolapse on straining and requirement for manual reduction (third degree); prolapsed and irreducible (fourth degree). Based on the 2024 guidelines from the American Society of Colon and Rectal Surgeons, hemorrhoids should be diagnosed clinically based on exam and history [44]. 

#### Treatment

Dietary modification is efficacious; increasing fiber and fluid intake have been studied extensively and are recommended by many societies [44]. Prolonged time on the toilet and straining have been shown to associated with hemorrhoids [45]. Topical medications, suppositories, and Sitz baths are beneficial for symptom relief with minimal risk [3, 44]. Rubber band ligation is the most effective in-office treatment for grade I, II, and III hemorrhoids that are refractory to conservative measures, and excisional hemorrhoidectomy should be offered for patients with grade III and IV hemorrhoids [44]. 

#### Prevention

There is little guidance on preventing hemorrhoids during pregnancy or the postpartum period. Topical hydrocortisone-pramoxine foam provided relief of symptoms and was shown to be safe for the fetus [46, 47]. High fiber diet, adequate fluid intake, and proper toileting behaviors can be helpful based on expert opinion [3, 9, 48]. 

### Pelvic Organ Prolapse

Pelvic organ prolapse (POP) is characterized by protrusion of a vaginal compartment or several compartments (anterior, posterior, apical) and the stage of prolapse is diagnosed clinically by lowest point of prolapse relative to the hymen on a POP quantitation index (POP-Q) [1]. Anatomic “prolapse” is highly prevalent in women, though exact numbers are not known [49]. Most importantly, however, women can be asymptomatic with POP; in a study of 477 women, 51% had prolapse to the level of the hymenal remnant or beyond without symptoms [50]. However, true prevalence of symptomatic POP is limited by reporting, and many women will not mention POP, or symptoms related to it, if they are not asked [49]. Surgery for POP is common; prevalence is estimated between 6 and 18% for women in their lifetime [51]. The decision to perform surgery for prolapse should be driven by the degree of bother.

The main risk factor for POP is vaginal childbirth. One study of 284 nulliparous women found that 25 developed POP at 6 weeks postpartum. The major risk factors were anatomical– such as levator ani distensibility, anterior position of the vaginal wall, distance between the urethra and the anus. Delivery route and major perineal injuries were not associated with POP; in fact none of the patients in the POP group had OASIS [52]. However, a major limitation of this study is its short follow-up period. Other studies have demonstrated strength of the pelvic floor and anatomic factors to be associated with POP, in addition to mode of delivery [1, 53]. Cesarian section is associated with a stronger pelvic floor and less POP in an extended postpartum period in these studies [1, 53]. In a longitudinal study of over 1,100 women 5–10 years after first vaginal birth, spontaneous vaginal birth (and especially operative vaginal birth) was associated with a significantly higher risk of development of POP when compared with Cesarean delivery [54]. 

#### Diagnosis

Diagnosis of POP is clinical, based on history and physical exam. History taking should utilize validated questionnaires, such as the PFDI-20 and PFIQ-7 [14, 51]. Physical exam, which includes a pelvic exam and split speculum exam, should also utilize the Pelvic Organ Prolapse Quantification (POP-Q) staging system [26, 49, 51]. Though not routinely recommended, urodynamics or a cough test can play a role in surgical planning to unmask occult UI. Additional tests for surgical planning include anorectal dynamic imaging and anorectal manometry in the setting of posterior vaginal prolapse or FI [26, 49]. 

#### Treatment

Treatment of symptomatic POP includes conservative measures, such as observation and PFMT, as well as more interventional therapies, such as use of pessaries and surgery [49]. Lifestyle modifications, such as weight loss, treating constipation, avoiding straining, and heavy lifting, are recommended by the International Consultation on Incontinence, in addition to PFMT [26]. A retrospective study showed that 66% of symptomatic POP patients who opted for observation as management continued for 24 months whereas 34% opted for pessary or surgery, after worsening of symptoms [55]. 

Pessaries are removable silicone intravaginal devices placed in the vaginal canal to help support the prolapsed organs, returning them to a more normal anatomic position. The purpose is to alleviate symptoms of vaginal bulge. Patients are fitted with pessaries with a pelvic exam and usually are taught how to remove the pessary themselves [49]. The fit is tested with the patient changing position, simulating physical activities, and voiding, to ensure comfort and that the pessary is not expelled, and that the patient can void. Risks of pessary use are generally minor, and include vaginal bleeding and erosions if not properly managed. These are usually treated with short-term removal and topical estrogen if women are postmenopausal, lactating, or have vaginal atrophy [26, 49]. 

There are multiple surgical options for POP, broadly split into two categories: reconstructive and obliterative surgeries [26]. Reconstructive surgeries maintain the vaginal canal for the purposes of penetrative intercourse and can use native tissues (when done transvaginally) or synthetic materials such as mesh (when done in a minimally invasive fashion transabdominally). For patients interested in transvaginal surgery for advanced prolapse who do not desire to maintain the vaginal canal (older, no longer sexually active), obliterative surgery (colpocleisis) is an option [49]. 

When comparing vaginal, native tissue repairs such as uterosacral ligament and sacrospinous ligament suspensions, success rates are similar and upwards of 60% [56]. Yet, when comparing native tissue transvaginal repair with abdominal mesh augmentation repair of apical prolapse, composite failure rates– usually defined as a combination of anatomic outcomes, need for prolapse retreatment, and patient symptoms– of mesh-augmented repairs are much lower (43% for native tissue vs. 29% for transvaginal mesh at 36 months) [57]. In a large systematic review of 56 trials including 5954 women, women who underwent sacral colpopexy was found to have superior outcomes to vaginal procedures (such as sacrospinous colpopexy, uterosacral colpopexy and transvaginal mesh); mesh-augmentation was associated with a lower rate of recurrent vault prolapse (3.5% vs. 15.3%) [58]. However, this higher success rate must be balanced with a longer operative time and risk of mesh exposure. While the rate of success for colpocleisis is nearly 100%, the procedure is only viable for women who no longer desire penetrative intercourse [59]. 

#### Prevention

There is very little data on the rate of progression of POP. Hence, there are no evidence-based recommendations for prevention or decreasing the risks of progression of POP.

### Urinary Incontinence

Urinary incontinence (UI) in the postpartum period is multifactorial. Stress urinary incontinence (SUI) and urgency urinary incontinence (UUI), are the most common phenotypes [2, 60]. UI can begin during pregnancy and persist or develop postpartum. In a cohort of pregnant women in the third trimester who were assessed using the PFDI, 41.8% reported urinary symptom distress [61]. Though some patients improve over time, others may not, or may have recurrence; in a longitudinal study of 3763 women, a prevalence of persistent UI 12 years after birth was 37.9% [62]. Though UI is common, over-normalization of UI should be avoided [5]. 

#### Diagnosis

Diagnosing SUI based on classic symptoms can be easily confirmed during pelvic examination, by asking the patient to cough or Valsalva with a full bladder. If the provider observes urine leaking from the urethra during an office cough/Valsalva stress test, the diagnosis of SUI is confirmed. Urodynamic testing can also be performed to confirm a suspected diagnosis of SUI, however, based on the Value of Urodynamic Evaluation study, in women with uncomplicated SUI without significant prolapse or urinary urgency, if SUI is demonstrated on examination, additional urodynamic testing is not necessary before treatment [63]. UUI is diagnosed by history and ensuring no acute cystitis, a normal post-void residual and normal screening urinalysis. Urodynamic testing may be considered if there are new-onset urinary symptoms and concern for pelvic nerve injury, such as in the setting of a prolonged second stage of labor or operative vaginal delivery. In women who have suspected bladder overdistension injury, urodynamics could help with diagnosis and determining prognosis, however, they are not mandatory [2]. 

#### Treatment

First line treatment for UI is PFMT, including Kegel contractions for SUI. PFMT can either be self-guided at home or done by physical therapist [2]. The American Urogynecologic Society and the International Urogynecological Association have patient resources available to guide home exercises online. In a recent systematic review, PFMT was noted to have significant benefit in treating UI [60]. Of 51 articles, only 8 met inclusion criteria, and 6/8 studies showed improvement. Another study compared low pressure fitness exercise, PFMT vs. minimal intervention/placebo for improving symptoms of PFD. Improvement in SUI was greatest in the low pressure fitness group [64]. Anti-incontinence intravaginal devices such as pessaries are also utilized for SUI and can be self-managed. Behavioral modifications, such as timed toileting and avoiding caffeine, are the primary treatments for UUI, in addition to PFMT [2]. Though there are newer, beta-adrenergic agonists approved for UUI (mirabegron, vibegron), they have not been studied in lactating women.

#### Prevention

PFMT during pregnancy has been studied as a method to prevent UI. A systematic review of 7 randomized controlled trials showed variable results [65]. Three studies did not have a statistically significant difference in UI. Additionally, SUI was assessed by self-reporting or surveys in all but one study (used a pad test with exercise). A Cochrane review revealed that antenatal PFMT slightly decreased the risk of UI in more than 3–6 months postpartum (29% less; RR 0.71, 95% CI 0.54 to 0.95) [16]. Given the low-risk nature of PFMT, the experts favor the use of PFMT for prevention of UI postpartum.

### Future Considerations for Research

#### Diagnostic Strategies

- Determine methods to improve rates of diagnosis of PFD: standardizing, validating, and universalizing questionnaires, both in the acute postpartum period and years after pregnancy.
- Streamline diagnostic pathways.

#### Treatment Strategies

- Study medications for constipation in pregnancy and postpartum, for safety and efficacy.
- Evaluate efficacy of translumbosacral neuromodulation for postpartum patients with FI, both in the acute period and in chronic persistent FI.

#### Preventative Strategies

- Determine individualized risk for PFD, to counsel on mode of delivery and anticipate complications based on modifiable and non-modifiable risk factors.
- Design large, high-quality trials to determine the role of exercise or PFMT for all postpartum PFD.
- Determine methods of prevention of PFD with subsequent pregnancies.

## Conclusions

Postpartum anorectal and PFD are common and can be very distressing. The prevalence, diagnosis, and management approaches for FI, constipation, hemorrhoids, POP, and UI are summarized in Table 2.

**Table 2:** Summary

Problem	Prevalence	Diagnosis	Treatment
Fecal and flatus incontinence	7 to 25%	Clinical history Rectal exam Anorectal manometry Anal ultrasound MR or barium defecography	High fiber diet, with fiber supplementation (goal 20–30 g/day) Lifestyle modification (e.g. timed toileting, avoiding caffeine) Pelvic floor muscle training Anal insert Vaginal-bowel control system Intrasphincter injection Vaginal electrical stimulation Sacral nerve stimulation Translumbosacral neuromodulation therapy Sphincteroplasty
Constipation	Up to 45%	Clinical history	Laxatives* High fiber diet, with fiber supplementation (goal 20–30 g/day)
Hemorrhoids	Up to 85%	Clinical history Digital rectal exam Anoscopy	High fiber diet, with fiber supplementation (goal 20–30 g/day) Behavioral modification (avoiding straining and prolonged time on the toilet) Topical therapies (hydrocortisone cream 1% or 2.5%, hydrocortisone suppository 25–30 g once or twice a day) Sitz baths Banding Excisional hemorrhoidectomy
Pelvic organ prolapse	6–18%	Clinical history Pelvic Floor Distress Inventory and Pelvic Floor Impact Questionnaire (PFDI-20 and PFIQ-7) Speculum and split speculum exam Urodynamics Dynamic imaging of the anorectum	Observation Pelvic floor muscle training Pessary Reconstructive surgery Obliterative surgery
Urinary incontinence	40%	Clinical history Speculum exam with cough test Urodynamics Urinalysis	Pelvic floor muscle training Pessary

*No specific laxatives are recommended postpartum based on available data. Osmotic laxatives such as polyethylene glycol 17g daily would be a logical first line therapy

## Electronic Supplementary Material

Below is the link to the electronic supplementary material.

Supplementary Material 1

## Data Availability

No datasets were generated or analysed during the current study.

## Key References

- Madsen AM, Hickman LC, Propst K. Recognition and Management of Pelvic Floor Disorders in Pregnancy and the Postpartum Period. Obstetrics and Gynecology Clinics of North America. 2021;48(3):571–84.
  - Detailed overview from the urogynecologist perspective of all pelvic floor disorders in pregnancy and postpartum.
- Rao SSC, Qureshi WA, Yan Y, Johnson DA. Constipation, Hemorrhoids, and Anorectal Disorders in Pregnancy. Am J Gastroenterol. 2022;117(10 S):16–25.
  - Comprehensive article from the gastroenterologist perspective with a focus on pregnancy.
- Meekins AR, Siddiqui NY. Diagnosis and Management of Postpartum Pelvic Floor Disorders. Obstetrics and Gynecology Clinics of North America. 2020;(3):477–86.
  - Thorough overview of all postpartum pelvic floor disorders.
