# Supplementary material for: Postpartum Anorectal and Pelvic Floor Disorders: Evaluation, Treatment, and Prevention
Source: Curr Gastroenterol Rep. 2025 Jul 3;27(1):48. doi: 10.1007/s11894-025-01000-7 (PMC12226706; doi:10.1007/s11894-025-01000-7)
Supplement: Supplementary file 1 — Supplementary Material 1 [file 11894_2025_1000_MOESM1_ESM.pdf]

PFDI- 20 Instructions: Please answer all of the questions in the following survey. These questions will ask you if you have certain bowel, bladder, or pelvic symptoms and, if you do, how much they bother you. Answer these by circling the appropriate number. While answering these questions, please consider your symptoms over the **last 3 months**.

The PFDI-20 has 20 items and 3 scales of your symptoms.
All items use the following format with a response scale from 0 to 4.

**Symptoms Present = YES, scale of bother**:	1 = not at all
	2 = somewhat
	3 = moderately
	4 = quite a bit
**Symptoms Not Present = NO**	0 = not present

Pelvic Organ Prolapse Distress Inventory 6 (POPDI-6)

Do you ...	No	Yes
1. Usually experience pressure in the lower abdomen?	0	1 2 3 4
2. Usually experience heaviness or dullness in the pelvic area?	0	1 2 3 4
3. Usually have a bulge or something falling out that you can see or feel in your vaginal area?	0	1 2 3 4
4. Ever have to push on the vagina or around the rectum to have or complete a bowel movement?	0	1 2 3 4
5. Usually experience a feeling of incomplete bladder emptying?	0	1 2 3 4
6. Ever have to push up on a bulge in the vaginal area with your fingers to start or complete urination?	0	1 2 3 4

Colorectal-Anal Distress Inventory 8 (CRAD-8):

Do you ...	No	Yes
7. Feel you need to strain too hard to have a bowel movement?	0	1 2 3 4
8. Feel you have not completely emptied your bowels at the end of a bowel movement?	0	1 2 3 4
9. Usually lose stool beyond your control if your stool is well formed?	0	1 2 3 4
10. Usually lose stool beyond your control if your stool is loose?	0	1 2 3 4
11. Usually lose gas from the rectum beyond your control?	0	1 2 3 4
12. Usually have pain when you pass your stool?	0	1 2 3 4
13. Experience a strong sense of urgency and have to rush to the bathroom to have a bowel movement?	0	1 2 3 4
14. Does part of your bowel ever pass through the rectum and bulge outside during or after a bowel movement?	0	1 2 3 4

Urinary Distress Inventory 6 (UDI-6):

Do you ...	No	Yes
15. Usually experience frequent urination?	0	1 2 3 4
16. Usually experience urine leakage associated with a feeling of urgency, that is, a strong sensation of needing to go to the bathroom?	0	1 2 3 4
17. Usually experience urine leakage related to coughing, sneezing, or laughing?	0	1 2 3 4
18. Usually experience small amounts of urine leakage (that is, drops)?	0	1 2 3 4
19. Usually experience difficulty emptying your bladder?	0	1 2 3 4
20. Usually experience *pain* or *discomfort* in the lower abdomen or genital region?	0	1 2 3 4

Scoring the PFDI-20:

Scale Scores: Obtain the mean value of all of the answered items within the corresponding scale (possible value 0 to 4) and then multiply by 25 to obtain the scale score (range 0 to 100). Missing items are dealt with by using the mean from answered items only.

PFSI-20 Summary Score: Add the scores from the 3 scales together to obtain the summary score (range 0 to 300).

PFIQ – 7 Instructions: Some women find that bladder, bowel, or vaginal symptoms affect their activities, relationships, and feelings. For each question place an **X** in the response that best describes how much your activities, relationships, or feelings have been affected by your bladder, bowel, or vaginal symptoms or conditions **over the last 3 months**. Please make sure you mark an answer in all 3 columns for each question.

How do symptoms or conditions relating to the following → → → Usually affect your …↓	***Bladder or urine***	***Bowel or rectum***	***Vagina or pelvis***
1. Ability to do household chores (cooking, housecleaning, laundry)?	☐ Not at all<br>☐ Somewhat<br>☐ Moderately<br>☐ Quite a bit	☐ Not at all<br>☐ Somewhat<br>☐ Moderately<br>☐ Quite a bit	☐ Not at all<br>☐ Somewhat<br>☐ Moderately<br>☐ Quite a bit
2. Ability to do physical activities such as walking, swimming, or other exercise?	☐ Not at all<br>☐ Somewhat<br>☐ Moderately<br>☐ Quite a bit	☐ Not at all<br>☐ Somewhat<br>☐ Moderately<br>☐ Quite a bit	☐ Not at all<br>☐ Somewhat<br>☐ Moderately<br>☐ Quite a bit
3. Entertainment activities such as going to a movie or concert?	☐ Not at all<br>☐ Somewhat<br>☐ Moderately<br>☐ Quite a bit	☐ Not at all<br>☐ Somewhat<br>☐ Moderately<br>☐ Quite a bit	☐ Not at all<br>☐ Somewhat<br>☐ Moderately<br>☐ Quite a bit
4. Ability to travel by car or bus for a distance greater than 30 minutes away from home?	☐ Not at all<br>☐ Somewhat<br>☐ Moderately<br>☐ Quite a bit	☐ Not at all<br>☐ Somewhat<br>☐ Moderately<br>☐ Quite a bit	☐ Not at all<br>☐ Somewhat<br>☐ Moderately<br>☐ Quite a bit
5. Participating in social activities outside your home?	☐ Not at all<br>☐ Somewhat<br>☐ Moderately<br>☐ Quite a bit	☐ Not at all<br>☐ Somewhat<br>☐ Moderately<br>☐ Quite a bit	☐ Not at all<br>☐ Somewhat<br>☐ Moderately<br>☐ Quite a bit
6. Emotional health (nervousness, depression, etc.)?	☐ Not at all<br>☐ Somewhat<br>☐ Moderately<br>☐ Quite a bit	☐ Not at all<br>☐ Somewhat<br>☐ Moderately<br>☐ Quite a bit	☐ Not at all<br>☐ Somewhat<br>☐ Moderately<br>☐ Quite a bit
7. Feeling frustrated?	☐ Not at all<br>☐ Somewhat<br>☐ Moderately<br>☐ Quite a bit	☐ Not at all<br>☐ Somewhat<br>☐ Moderately<br>☐ Quite a bit	☐ Not at all<br>☐ Somewhat<br>☐ Moderately<br>☐ Quite a bit

All of the items use the following response scale:

0 = not at all; 1 = somewhat, 2 = moderately, 3 = quite a bit

Scales:

Urinary Impact Questionnaire (UIQ-7); 7 items under column heading “Bladder or urine.”
Colorectal-Anal Impact Questionnaire (CRAIQ-7): 7 items under column heading “Bowel or rectum.”
Pelvic Organ Prolapse Impact Questionnaire (POPIQ-7): 7 items under column heading “Pelvis or vagina.”

<u>Scale scores</u>: Obtain the mean value for all of the answered items within the corresponding scale (possible value 0 to 3) and then multiply by 100/3) to obtain the scale score (range 0 to 100). Missing items are dealt with by using the mean from answered items only.

Total score of each section ________ divided by 7 ______ X 33.3 = _________

<u>PFIQ-7 Summary Score</u>: Add the scores from the 3 scales together to obtain the summary score (range 0 to 300).

Barber, M., Walters, M., et al. (2005). "Short forms of two condition-specific quality of life questionnaires for women with pelvic floor disorders (PFDI-20 and PFIQ -7)." American Journal of Obstetrics and Gynecology 193: 103-113.

Pelvic Floor Impact Questionnaire—short form 7 (PFIQ-7)

Name______________________ DATE______________

DOB______________________

Instructions: Some women find that bladder, bowel, or vaginal symptoms affect their activities, relationships, and feelings. For each question, check the response that best describes how much your activities, relationships, or feelings have been affected by your bladder, bowel, or vaginal symptoms or conditions **over the last 3 months.** Please make sure you mark an answer in **all 3 columns** for each question.

How do symptoms or conditions in the following usually affect your	***Bladder or urine***	***Bowel or rectum***	***Vagina or pelvis***
1. Ability to do household chores (cooking, laundry housecleaning)?	□Not at all<br>□Somewhat<br>□Moderately<br>□Quite a bit	□Not at all<br>□Somewhat<br>□Moderately<br>□Quite a bit	□Not at all<br>□Somewhat<br>□Moderately<br>□Quite a bit
2. Ability to do physical activities such as walking, swimming, or other exercise?	□Not at all<br>□Somewhat<br>□Moderately<br>□Quite a bit	□Not at all<br>□Somewhat<br>□Moderately<br>□Quite a bit	□Not at all<br>□Somewhat<br>□Moderately<br>□Quite a bit
3. Entertainment activities such as going to a movie or concert?	□Not at all<br>□Somewhat<br>□Moderately<br>□Quite a bit	□Not at all<br>□Somewhat<br>□Moderately<br>□Quite a bit	□Not at all<br>□Somewhat<br>□Moderately<br>□Quite a bit
4. Ability to travel by car or bus for a distance greater than 30 minutes away from home?	□Not at all<br>□Somewhat<br>□Moderately<br>□Quite a bit	□Not at all<br>□Somewhat<br>□Moderately<br>□Quite a bit	□Not at all<br>□Somewhat<br>□Moderately<br>□Quite a bit
5. Participating in social activities outside your home?	□Not at all<br>□Somewhat<br>□Moderately<br>□Quite a bit	□Not at all<br>□Somewhat<br>□Moderately<br>□Quite a bit	□Not at all<br>□Somewhat<br>□Moderately<br>□Quite a bit
6. Emotional health (nervousness, depression, etc)?	□Not at all<br>□Somewhat<br>□Moderately<br>□Quite a bit	□Not at all<br>□Somewhat<br>□Moderately<br>□Quite a bit	□Not at all<br>□Somewhat<br>□Moderately<br>□Quite a bit
7. Feeling frustrated?	□Not at all<br>□Somewhat<br>□Moderately<br>□Quite a bit	□Not at all<br>□Somewhat<br>□Moderately<br>□Quite a bit	□Not at all<br>□Somewhat<br>□Moderately<br>□Quite a bit

Total x 100 x 100 x 100
Scoring the PFIQ-7: =
All of the items use the following response scale:
0, Not at all; 1, somewhat; 2, moderately; 3, quite a bit **PFIQ-7 Score Scales:**
Urinary Impact Questionnaire (UIQ-7): 7 items under column heading “Bladder or urine”
Colorectal-Anal Impact questionnaire (CRAIQ-7): 7 items under column heading “Bowel / rectum”
Pelvic Organ Prolapse Impact Questionnaire (POPIQ-7): Items under column “Pelvis / Vagina”
Scale Scores: Obtain the mean value for all of the answered items within the corresponding scale (possible value 0 – 3) and then multiply by (100/3) to obtain the scale score (range 0-100).
Missing items are dealt with by using the mean from answered items only.
PFIQ-7 Summary Score: Add the scores from the 3 scales together to obtain the summary score (range 0-300).
